# 

**Published:** 1882-12

**Authors:** 


December, 1882.

Number 6, Vol. XLV.

### THE CHICAGO MEDICAL Journal and Examiner

EDITORS:

N. S. DAVIS, M.D., LL.D. JAS. NEVINS HYDE, A.M., M.D.

DANIEL R. BROWER, M.D.

CHICAGO MEDICAL PRESS ASSOCIATION.

AUG. 2D

1875.

CHICAGO:

*PUBLISHED BY THE CHICAGO MEDICAL PRESS ASSOCIATION,*

188 Clark Street.

☞ Read the Notice of this Journal among Advertisements

